# Family history recording in UK general practice: the lIFeLONG study

**DOI:** 10.1093/fampra/cmab117

**Published:** 2021-09-27

**Authors:** Molly Dineen, Kate Sidaway-Lee, Denis Pereira Gray, Philip H Evans

**Affiliations:** College of Medicine and Health, University of Exeter, Exeter, Devon, United Kingdom; St Leonard’s Practice, Exeter, Devon, United Kingdom; St Leonard’s Practice, Exeter, Devon, United Kingdom; College of Medicine and Health, University of Exeter, Exeter, Devon, United Kingdom; St Leonard’s Practice, Exeter, Devon, United Kingdom; College of Medicine and Health, University of Exeter, Exeter, Devon, United Kingdom; St Leonard’s Practice, Exeter, Devon, United Kingdom

**Keywords:** family history, family medicine, family practice, general practice, genomic medicine, medical records

## Abstract

**Background:**

In order to integrate genomic medicine into routine patient care and stratify personal risk, it is increasingly important to record family history (FH) information in general/family practice records. This is true for classic genetic disease as well as multifactorial conditions. Research suggests that FH recording is currently inadequate.

**Objectives:**

To provide an up-to-date analysis of the frequency, quality, and accuracy of FH recording in UK general/family practice.

**Methods:**

An exploratory study, based at St Leonard’s Practice, Exeter—a suburban UK general/family practice. Selected adult patients registered for over 1 year were contacted by post and asked to complete a written FH questionnaire. The reported information was compared with the patients’ electronic medical record (EMR). Each EMR was assessed for its frequency (how often information was recorded), quality (the level of detail included), and accuracy (how closely the information matched the patient report) of FH recording.

**Results:**

Two hundred and forty-one patients were approached, 65 (27.0%) responded and 62 (25.7%) were eligible to participate. Forty-three (69.4%) EMRs contained FH information. The most commonly recorded conditions were bowel cancer, breast cancer, diabetes, and heart disease. The mean quality score was 3.64 (out of 5). There was little negative recording. 83.2% of patient-reported FH information was inaccurately recorded or missing from the EMRs.

**Conclusion:**

FH information in general/family practice records should be better prepared for the genomic era. Whilst some conditions are well recorded, there is a need for more frequent, higher quality recording with greater accuracy, especially for multifactorial conditions.

**Key Messages**

- Research suggests that FH recording in general/family practice is inadequate.
- This study assessed the current frequency, quality, and accuracy of FH recording.
- For some conditions there was frequent FH recording.
- There were major gaps in recording, particularly for negative FH information.
- There was limited recording of multifactorial conditions.
- More research is needed on the barriers to FH recording to facilitate its use.

## Background

Genomic science is offering the potential to further personalize medicine.^1,2^ All specialties must be prepared with the resources, knowledge, and confidence to incorporate the advances in genomic medicine, including general/family practice.

A relatively quick, cost-effective, and practical method of gaining an insight into a patient’s genomic information^3,4^ can be taking a family history (FH). General practitioners (GPs; family physicians) are in an optimal position to gather FH information due to their holistic viewpoint, frequent consultation rate, and commonly being the first point of contact for multiple family members.^5,6^ In order to integrate genomics into routine patient care and stratify personal risk, GPs must frequently, thoroughly, and accurately record FH information.^4^ This is true for classical genetic disorders but also for conditions or traits that are caused by a combination of multiple genomic and environmental factors, such as depression or obesity.^7–13^ As defined in Health Education England’s Genomics Education Programme, these conditions are hereafter referred to as “multifactorial conditions.”^13^

Despite significant efforts (mainly in the United States) to develop digitized collection tools,^14–18^ research suggests that the standard of FH recording in general/family practice is currently inadequate.^4,19^ GPs are acknowledging the importance of genomic medicine and are willing to learn,^5,20^ but are still lacking validated tools, educational interventions, and standardized protocols to operationalize its use in every day practice.^3,8,21,22^

This study aimed to evaluate the current scale of the problem and build upon existing evidence to specifically identify the areas that require improvement. This was achieved by reviewing the FH information recorded in the electronic medical records (EMRs) of a single UK general/family practice. This was the first study of its kind to assess FH recording for a number of multifactorial conditions.

## Methods

This exploratory study compared the recording of FH information in patients’ EMRs with up-to-date patient reports. Similar methodology had been employed in previous studies.^10,19,23^ We defined our own three discrete measures to subcategorize the standard of FH recording. The *frequency* of FH recording, how often any FH information was recorded—positive or negative.^24^ The *quality* of recording, the level of detail included when recording a positive FH (PFH).^19,25^ And the *accuracy* of recording, how closely the recorded information matched the patients’ reports.^3^

The study was based at St Leonard’s Practice (SLP), Exeter, UK—a suburban general/family practice in Southwest England with approximately 9,300 patients and 8 part-time GPs. The practice uses the EMR system, SystmOne, which is used by more than 2,700 general practices across England.^26^

Eligible patients were over 18, registered at SLP for at least 1 year. Patients were randomly selected using SystmOne. The sample size was determined by feasibility, as due to the exploratory nature of the study and the limited number of comparable studies, it was not possible to conduct a sample size calculation.

Patient names were screened by their own GP. Patients were excluded if they were considered unable to complete a FH questionnaire (FHQ) in written English, did not have the capacity to consent (e.g. due to a learning disability) or were inappropriate to approach (e.g. terminally ill). Eligible patients were posted information packs, including a FHQ and return envelope. One reminder was sent.

Patients were asked to complete a validated 12-item FHQ,^27^ adapted to gather more information (e.g. age of diagnosis) and extended to include nine additional multifactorial conditions (see Supplementary Fig. S1). The information given was considered to be accurate and up-to-date as studies have suggested that patient-reported FH information is a valid comparator.^12^

Each patient’s EMR was manually reviewed by a member of the research team (MD) for FH Read codes (RCs). RCs (now SNOMED codes) are a hierarchical coded thesaurus of clinical terms which the GP can attach to a patient’s EMR at any time, with descriptive free text, e.g. (1252.) FH: Diabetes mellitus.^28^ FH RCs automatically appear on the SystmOne summary screen which displays the most pertinent patient information when first accessing a record. As in a similar study^10^ and in large databases (e.g. Clinical Practice Research Datalink), free text and non-FH RCs were not included as this information was less readily available for a working GP. Recordings related to a spouse/partner and duplicate recordings were not included.

To determine frequency, each EMR was marked as having PFH information, negative FH (NFH) information or no FH information for each condition. The proportions of patients with a self-reported PFH and a recorded PFH were compared using a comparison of proportions test.

In the absence of a standard scoring system, a categorical quality score was devised de novo based on the literature and a score was assigned to each PFH recording in the EMR. The score was out of five, as each element required for accurate risk assessment achieved a score of one^29^—condition, relative, lineage (maternal or paternal), gender, and age of diagnosis. When a single RC referenced multiple relatives, recordings were scored individually. This method of quality scoring was validated in a 10% sample by a second member of the research team (PHE).

In each case of a patient-reported PFH, the EMR was marked as accurate, moderately accurate, or inaccurate. It was considered accurate if it matched the information in the FHQ, moderately accurate if the PFH status was recorded but with inaccurate detail (e.g. not all affected relatives noted) and inaccurate if it entirely missed or contradicted the information in the FHQ.

All FH information missing from the EMR was added at the end of the study and the patients’ own GP was alerted about clinically important information.

All extracted data were entered into Microsoft Excel (Microsoft, version 14, 2010) and were analyzed using Stata SE (Stata, version 15.0, 2017). Significance was taken as a *P* value of <0.05.

This study took place from March 2019 to July 2019.

## Results

Two hundred and fifty-two patients were randomly selected (3.8% of the practice’s population of over 18s). Eleven patients were excluded, and 241 patients were sent invitation packs. Of these, 65 (27.0%) patients responded and 62 (25.7%) were willing and eligible to participate (see Supplementary Fig. S2). The participants’ demographics are reported in Table 1. The mean age of the sample was 60.81 years (95% confidence interval [CI]: 60.44–61.17) and 48.98 (95% CI: 48.55–49.41) in the practice’s adult population. The median age was 48 years (interquartile range [IQR] 35–63) and 47 (IQR 34–62) in the practice’s adult population. The mean number of years registered was 14.54 years (95% CI: 14.25–14.83) and 15.4 (95% CI: 15.01–15.74) in the practice’s adult population.

**Table 1.** Demographic characteristics of the 62 patients in the study sample with a comparison to the 6,684 patients in the practice’s adult population (2019).

Characteristic	Number of patients (*n* (%))	For comparison, the adult patient population registered at SLP for >1 year (*n* (%))
Age		
20–40 years	9 (14.5)	2,417 (36.2)
41–60 years	17 (27.4)	2,318 (34.7)
61–80 years	29 (46.8)	1,483 (22.2)
>80 years	7 (11.3)	343 (5.13)
Sex		
Male	24 (38.7)	3,204 (47.9)
Female	38 (61.3)	3,480 (52.1)
Years registered with SLP		
<20 years	45 (72.6)	4,771 (71.4)
>20 years	17 (27.4)	1,913 (28.6)
Total	62 (100)	6,684 (100)

### The frequency of FH recording

Forty-three of the 62 patients (69.4%) had some FH information recorded in their EMR. In total, there were 129 FH RCs entered—a mean of 2.08 RCs (95% CI: 1.57–2.60) per patient. Twelve of these RCs were excluded from the analysis as they were either invalidated by a subsequent recording (*n* = 3) or were considered incorrect as the descriptive text contradicted its RC (*n* = 9). This left 117 valid RCs for the 62 patients—a mean of 1.89 FH RCs per patient (95% CI: 1.43–2.44).

Forty of these 62 patients (64.5%) patients had PFH information recorded and 10 (16.1%) had NFH information recorded.

The frequency of recording for each condition in the original FHQ can be seen in Table 2. The most commonly recorded conditions were bowel cancer, breast cancer, diabetes, and heart disease. For these conditions, there was no significant difference between the proportion of patients with a self-reported PFH and the proportion of patients with a PFH in their notes. There was no FH recording for any of the additional multifactorial conditions. For these conditions, there was a significant difference (*P* values all < 0.05) between the proportion of patients with a self-reported PFH (Table 3) and the proportion of patients with a recorded PFH, other than for drug addiction and autism for which the numbers of patient-reported PFH were low.

**Table 2.** The frequency of FH recording in the EMRs of 62 patients in general/family practice by condition (original conditions arranged in the order they appear in the adapted FHQ-12) (2019).

Condition (or characteristic)	Patients with a self-reported PFH (*n* (% of all patients))	Frequency of recording (*n* (% of all patients))		
		Patients with a PFH recorded	Patients with a NFH recorded	Patients with no FH info. recorded
Premature heart disease (<60 years)	21 (33.9)	25 (40.3)	5 (8.1)	31 (50.0)
Type 2 diabetes mellitus	10 (16.1)	6 (9.7)	1 (1.6)	54 (87.1)
SE Asian ancestry	2 (3.2)	0 (0)	0 (0)	62 (100)
Indian ancestry	1 (1.6)	0 (0)	0 (0)	62 (100)
Early onset prostate cancer	3 (4.8)	1 (1.6)	0 (0)	61 (98.4)
Ovarian cancer	4 (6.5)	0 (0)	0 (0)	62 (100)
Early onset breast cancer	11 (17.7)	8 (12.9)	0 (0)	54 (87.1)
Jewish ancestry	1 (1.6)	0 (0)	0 (0)	62 (100)
Eastern or Central European ancestry	1 (1.6)	0 (0)	0 (0)	62 (100)
Early onset bowel cancer (<55 years)	7 (11.3)	6 (9.7)	0 (0)	56 (90.3)
Other (see Supplementary Fig. S3)	26 (41.9)	24 (38.7)	7 (11.3)	33 (53.2)

**Table 3.** The number of patients self-reporting a PFH by condition (additional multifactorial conditions arranged in the order they appear in the adapted FHQ-12) (2019).

Condition or characteristic	Patients with a self-reported PFH (*n* (% of all patients))
Obesity	18 (29.0)
Suicide	5 (8.1)
Alcoholism	6 (9.7)
Psychosis	5 (8.1)
Drug addiction	1 (1.6)
Depression	19 (30.6)
Anxiety	19 (30.6)
Migraine	16 (25.8)
Autism	2 (3.2)

### The quality of FH recording

The 111 positive entries were scored for quality. The mean quality score was 3.64 out of 5 (95% CI: 3.42–3.86). One hundred and five of 111 positive entries (94.6%) recorded the condition, 96 (86.5%) recorded the relative, 85 (76.6%) recorded lineage, 97 (87.4%) recorded gender, and 21 (18.9%) recorded age of disease onset or death. The mean quality scores for each condition can be seen in Table 4. When validated by a second member of the research team, the level of agreement between the scores assigned was substantial, indicated by a Kappa of 0.78.^30^

**Table 4.** The quality of FH recording in the EMRs of 62 patients in general/family practice, scored out of a possible 5, by condition (conditions with PFH recorded, arranged in descending order of quality^a^) (2019).

Condition or characteristic	Quality of recording (max score 5)
	Mean quality score (95% CI)
Early onset bowel cancer	4.14 (3.86–4.42)
Early onset prostate cancer	4.0 (actual value)
Early onset breast cancer	3.78 (3.34–4.21)
Premature heart disease	3.73 (3.32–4.14)
Other (see Supplementary Fig. S3)	3.51 (3.15–3.87)
Type 2 diabetes mellitus	3.30 (2.71–3.89)

^a^Conditions with no FH recording in the sampled EMRs were omitted.

### The accuracy of FH recording

There were 185 cases of self-reported PFH in 55 patients—a mean of 2.98 (95% CI: 2.50–3.47) per patient in the total sample. Fifteen (8.1%) of these were accurately recorded in the EMR, 12 (6.5%) were moderately accurately recorded, and 154 (83.2%) were inaccurately recorded. The accuracy of recording for each condition can be seen in Table 5.

**Table 5.**
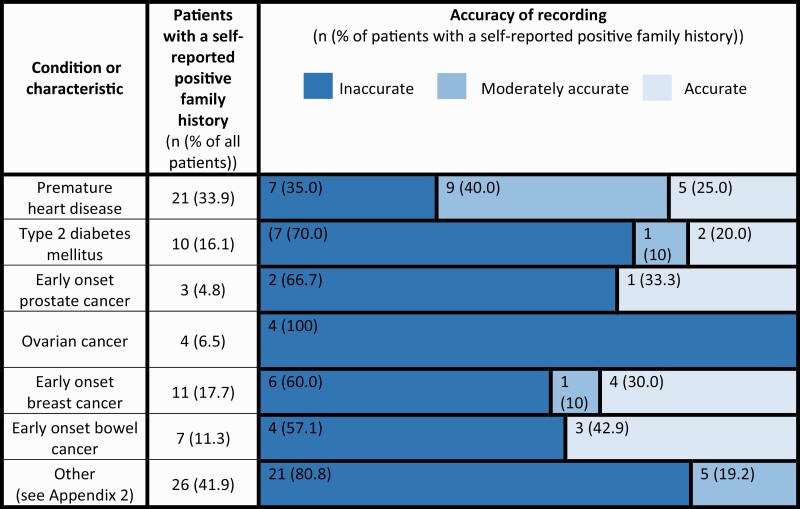
The accuracy of FH recording in the EMRs of 62 patients in general/family practice by condition (conditions with PFH recorded, arranged in descending order of quality^a^) (2019).

^a^Conditions with no FH recording in the sampled EMRs were omitted. Ancestry was omitted for the same reason.

## Conclusions

### Summary

Of the 62 patient records analyzed there was a high frequency of FH recording with 69.4% of records containing FH information. Recordings were primarily of PFH as there was limited use of NFH RCs. The most commonly recorded conditions were bowel cancer, breast cancer, diabetes, and heart disease. There was no recording for any of the additional multifactorial conditions. The mean quality score was 3.64 out of 5. Relative, lineage, and gender were well recorded but age of disease onset or death less so. Overall, the accuracy of recording was poor, with 83.2% of patient-reported PFH information inaccurately recorded, or missing from the EMRs.

### Strengths and limitations

This study provided an up-to-date insight into FH recording in a working UK general/family practice. The results may not be generalizable to other practices as they may be dependent on individual clinician enquiry and the recording behavior of the practice. However, there are many good, recent examples of exploratory single-practice studies^31^ and here, a single-practice was suitable for initial in-depth analysis.

The sample of patients who responded was not fully representative of the adult patient population at SLP. There was a higher proportion of women in the sample, which may be associated with a higher level of FH recording.^25^ On average, the patients sampled were also older, making them more likely to have PFH. It is possible that there was an element of responder bias and that patients with significant FH were more likely to respond and more likely have reported this information to their GP. Unfortunately, a sample size calculation was not possible, and the sample was relatively small; however, this was the first attempt at scoping this type of data in detail. The response rate of 25.3% was similar to Qureshi et al. who had a response rate of 10.7% after also posting FHQs to patients.^32^

In this study, the information given by patients was considered to be accurate, but some research has suggested that patients’ knowledge of their FH is limited.^24^ The quality scores should be interpreted with caution, as the properties of the novel scoring system would need to be formally tested for its validity. However, in the absence of a standard scoring system, this assessment of quality was appropriate in this instance, particularly due to the scoping nature of the study. Free text was not explored and this may have contained important FH information. The methodology has the potential to be replicated on a larger scale and/or in other general/family practices to increase the generalizability and reliability of the findings.

### Comparison with existing literature

Just over two thirds (69.4%) of patients had FH information in their EMR. This is a relatively high frequency of recording when compared with similar research, other than one study which found 97% of EMRs contained FH information.^19^ Two recent studies found that 12% and 56.6% of primary care records contained FH information respectively,^10,32^ although the comparisons are limited by the differing settings. In the most comparable general/family practice setting, Rafi et al. found 39.3% of EMRs contained FH information.^33^

The most frequently recorded conditions were bowel cancer, breast cancer, diabetes, and heart disease. For premature heart disease, more patients had a PFH recorded than was self-reported. GPs may be more motivated to record a FH of heart disease due to its well-known predictive accuracy, the clear options for intervention and the introduction of risk scores, such as the QRISK2, which act as a recording tool.^25^ Despite the high frequency of recording for these conditions, key quality items, such as the age of disease onset or death, were often missing. This information has significant implications for clinical practice; guiding ongoing care and referral behavior.

Interestingly, there were no patients with recorded FH information for any of the additional multifactorial conditions, despite a significant number of patients reporting a PFH and despite the availability of relevant RCs for each condition. Nearly a third of patients reported a PFH of depression but no FH information was recorded in any of their notes. This was the first study of this kind to specifically assess the recording of these conditions. The low level of recording might show a subconscious bias toward physical medicine or a lack of understanding about their association with genomic medicine. The low frequency of recording also probably represents a limitation of the EMR system; we observed that the process of selecting RCs was cumbersome, due to the long list containing duplications and irrelevant codes. There were 12 instances when RCs were obviously used in error. However, there is a generic FH RC which, with a descriptor, can be used for any condition.

Only 16.1% of patients had any NFH information recorded despite the fact that NFH information is just as important as PFH information for the accurate risk assessment of disease.^5^ The infrequent use of negative recording is consistent with the literature. This is despite the availability of relevant negative RCs. Powell et al. in the United States found that 4.3% of 390 community practice records contained NFH information.^19^ It is unsurprising, as when interviewed on this subject in 2013, GPs stated that they rarely document a negative finding.^5^

Perhaps most significantly, only 8.1% of self-reported PFH information was accurately recorded in the EMR. 83.2% of this information was inaccurately recorded or missing from the patient record. The literature on this is limited, but one 2017 study in Canada, demonstrated that more patients were at risk as a result of their FH, than was reflected in the records.^23^ In this study, a lack of agreement between the EMR and the FHQ may have resulted from the specific limits given in the questionnaire (e.g. conditions, age of diagnosis) or from the obvious disparity between what patients and GPs consider relevant FH information—see Supplementary Figs. S3 and S4. The literature suggests the pertinent barriers to accurate FH recording are the lack of regular updating, the ever-changing nature of the FH and inaccurate patient recall.^4,5,24^

### Implications for research and/or practice

The FH in general practice has never been more important, with the new genomic knowledge available and the opportunity to gather this information.

This study demonstrated the potential that UK general/family practice has for frequent, high quality, and accurate FH recording. With some conditions well recorded, this study showed that FH recording in modern general/family practice is perfectly possible. However, generally, the EMRs in UK general/family practice are not adequately prepared for the genomic era; there is a lack of standardized recording practice which has resulted in significant gaps in the data. In this study, only 8.1% of self-reported PFH information was accurately recorded in the EMR. EMR systems must be developed to better facilitate recording. This could include an embedded FHQ for new patients, containing all quality items; the ability for patients to add information directly to their record; the requirement to code all FH information and/or incentivising FH recording.

Further development requires national policies to identify FH recording as a priority. This publication should alert both clinicians and educators, to galvanize new attention on FH recording and optimize patient care. Future research should usefully clarify the specific weaknesses in current practice and the barriers still preventing frequent, high quality, and accurate FH recording. Research should be conducted to address these barriers and develop specific interventions which will facilitate progress. GPs may be subconsciously biased toward recording the FH of physical conditions for which there is evident clinical utility, and therefore research and education around the clinical utility of the FH for the additional multifactorial conditions should also be a priority.

## Supplementary Material

cmab117_suppl_Supplementary_Figure_S1

cmab117_suppl_Supplementary_Figure_S2

cmab117_suppl_Supplementary_Figure_S3

cmab117_suppl_Supplementary_Figure_S4

cmab117_suppl_Supplementary_Checklist

## Data Availability

The data underlying this article are available in the article and in its online supplementary material.
