# Supplementary material for: Family history recording in UK general practice: the lIFeLONG study
Source: Fam Pract. 2021 Sep 27;39(4):610–5. doi: 10.1093/fampra/cmab117 (PMC9295608; doi:10.1093/fampra/cmab117)
Supplement: cmab117_suppl_Supplementary_Figure_S1 [file cmab117_suppl_supplementary_figure_s1.pdf]

Figure S1: The family history questionnaire adapted from Walter et al. [25] completed by patients in the lIFeLONG study (2019)

Family history questionnaire

Please take time to answer all questions in both parts of the questionnaire.

Part 1

	Question	Yes	No	If yes, please give details of which relative and at what age:
1	Do you think that there are any conditions or illnesses that run in your family? If so, please specify: _________			
2	Have any of your close relatives, including parent(s), children, brother(s), or sister(s), had heart disease (also known as cardiovascular disease, heart attack, angina, bypass surgery) before the age of 60?			
3	Have any of you close relatives, including parent(s), children, brother(s), or sister(s), had diabetes (also known as type 2 diabetes or non-insulin dependent diabetes)?			
4	Do you come from any of the following ancestry?<br>People from these backgrounds may be at increased risk of diabetes			
(a)	South East Asia			
(b)	Indian subcontinent, that is, India, Pakistan, Bangladesh			
5	Have any of your close male relatives, including father, son(s) or brother(s), had prostate cancer before the age of 60?			
6	Have any of your close female relatives, including mother, daughter(s) or sister(s), had ovarian cancer?			

7	Have any of your close relatives, including parent(s), children, brother(s), or sister(s), had breast cancer before the age of 50?			
8	Do you have more than one relative who has had breast cancer on either you mother's or father's side of the family?<br>Please think about all of the following relatives: parent(s), children, brother(s), sister(s), grandparents(s), aunt(s), uncle(s), niece(s), nephew(s), and grandchildren.			
9 (a)	Is your family of Jewish ancestry?			
9 (b)	If so, are they from Eastern or Central Europe (Ashkenazi)? People from these backgrounds may be at increased risk of breast cancer.			
10	Have any of your close relatives, including parent(s), children, brother(s), or sister(s), had colon or rectal cancer (also known as large bowel or colorectal cancer) before the age of 55?			
11	Do you have more than one relative who has had colon or rectal cancer on either your mother's or father's side of the family? Please think about all of the following relatives: parent(s), children, brother(s), sister(s), grandparents(s), aunt(s), uncle(s), niece(s), nephew(s), and grandchildren.			

Thank you for completing part 1 of the family history questionnaire. Please now continue overleaf to complete part 2.

12	Have any of your relatives been obese? This is also known as having a body mass index (BMI) over 30.			
13	Have any of your relatives died by suicide?			
14	Have any of your relatives had an alcohol dependency? This is also known as alcoholism or alcohol addiction.			
15	Have any of your relatives had psychosis? This will include schizophrenia and bi-polar/manic-depressive disorder.			
16	Have any of your relatives had a drug addiction? This is also known as drug dependency.<br>Please note, this does not include an alcohol addiction.			
17	Have any of your relatives had depression?			
18	Have any of your relatives had anxiety?			
19	Have any of your relatives had migraines?			
20	Have any of your relatives been diagnosed as autistic? This will include Asperger syndrome.			

Thank you for completing this questionnaire. Now, please return this **and your signed consent form** in the pre-paid envelope provided.
