# Supplementary figures and images for: Family history recording in UK general practice: the lIFeLONG study
Source: Fam Pract. 2021 Sep 27;39(4):610–5. doi: 10.1093/fampra/cmab117 (PMC9295608; doi:10.1093/fampra/cmab117)

Figure S2: IIFeLONG study recruitment flow chart (2019)

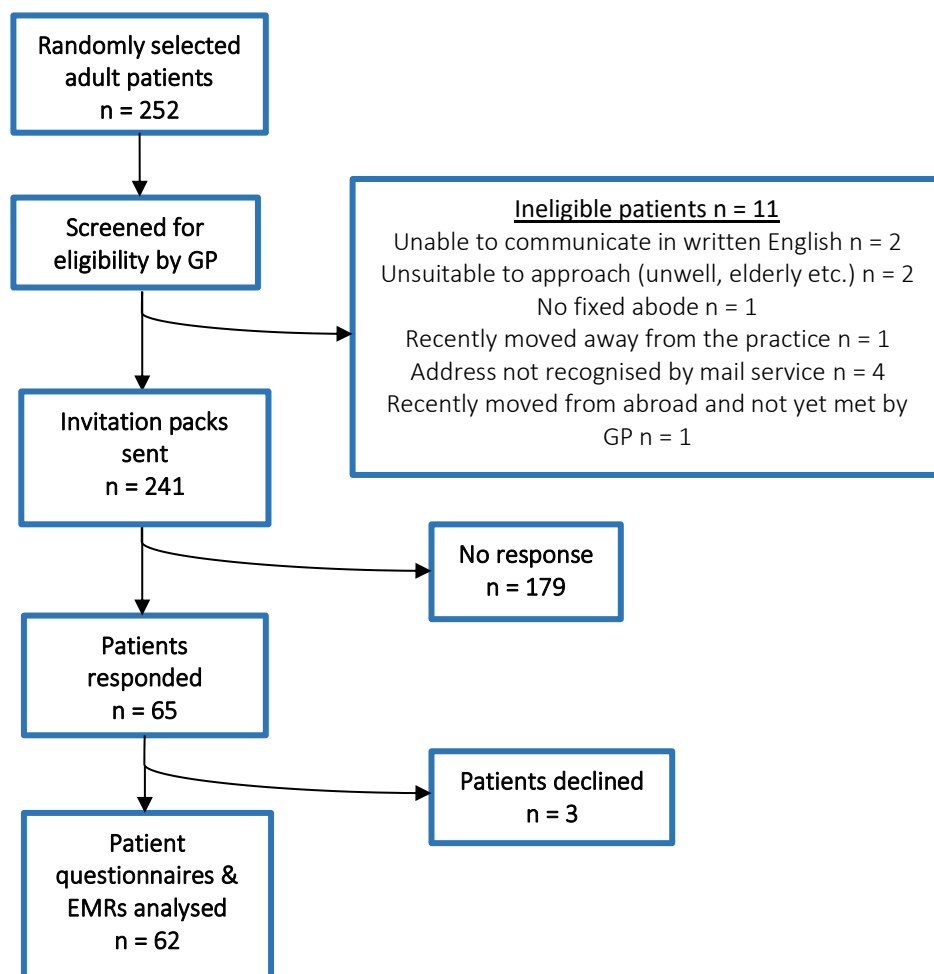

Supplement: cmab117_suppl_Supplementary_Figure_S2 [file cmab117_suppl_supplementary_figure_s2.pdf]
