# Supplementary material for: Family history recording in UK general practice: the lIFeLONG study
Source: Fam Pract. 2021 Sep 27;39(4):610–5. doi: 10.1093/fampra/cmab117 (PMC9295608; doi:10.1093/fampra/cmab117)
Supplement: cmab117_suppl_Supplementary_Figure_S3 [file cmab117_suppl_supplementary_figure_s3.pdf]

Figure S3: The conditions not listed in the lIFeLONG FHQ but included by patients when asked ‘Do you think that there are any conditions or illnesses that run in your family?’ (2019)

Ankylosing spondylitis
Arthritis
Asthma
B27 arthritis
Bronchiectasis
Cancer
Crohn’s disease
Coeliac
Dementia
Eczema
Glaucoma
Heart problems
Hereditary spastic paraparesis
Hiatus hernia
Hypercholesterolaemia
Hyperthyroidism
Hypothyroidism
Lactose intolerance
Lung conditions
Lupus
Muscular dystrophy
NHL
Reactive arthritis
Stroke
