# Supplementary material for: Family history recording in UK general practice: the lIFeLONG study
Source: Fam Pract. 2021 Sep 27;39(4):610–5. doi: 10.1093/fampra/cmab117 (PMC9295608; doi:10.1093/fampra/cmab117)
Supplement: cmab117_suppl_Supplementary_Figure_S4 [file cmab117_suppl_supplementary_figure_s4.pdf]

Figure S4: The conditions not listed in the lIFeLONG FHQ but with FH information recorded in the patients' electronic general practice records (2019)

Asthma
Brain tumour
Cancer
Cervical cancer
Collapsed lung
Epilepsy
Glaucoma
Hay fever
Hypercholesterolaemia
Leukaemia
Lung cancer
Pernicious anaemia
Skin and subcutaneous tissue disease
Stroke
Unknown condition
